# Utilizing Camera Traps, Closed Circuit Cameras and Behavior Observation Software to Monitor Activity Budgets, Habitat Use, and Social Interactions of Zoo-Housed Asian Elephants (*Elephas maximus*)

**DOI:** 10.3390/ani10112026

**Published:** 2020-11-03

**Authors:** Jilian M. Fazio, Tony Barthel, Elizabeth W. Freeman, Kay Garlick-Ott, Anne Scholle, Janine L. Brown

**Affiliations:** 1 Department of Animal Programs, Animal Care Sciences, Smithsonian’s National Zoological Park, 3100 Connecticut Ave., Washington, DC 20013, USA; barthelt@si.edu (T.B.); garlickottkj@gmail.com (K.G.-O.); anne.scholle@gmail.com (A.S.); 2 Center for Species Survival, Smithsonian’s Conservation Biology Institute, 1500 Remount Rd., Front Royal, VA 22630, USA; brownjan@si.edu; 3 School of Integrative Studies, George Mason University, 4400 University Dr, Fairfax, VA 22030, USA; efreeman@gmu.edu

**Keywords:** animal welfare, Asian elephant, ex-situ management, ZooMonitor, camera traps

## Abstract

**Simple Summary:**

Zoological institutions place strong emphasis on monitoring the welfare of individual animals in their care. Long-term behavior data can be integrated into zoological management to allow for adjustments on a continuous basis as animals respond over time to social, environmental, or physical changes. Behavioral observations conducted for two years on one male and six female Asian elephants (*Elephas maximus*) using Zoo Monitor, a closed-circuit camera system, and camera traps revealed individual activity budgets, habitat use and social interactions in ways beyond use of these tools in isolation. This multi-tool approach provides a broader assessment of individual welfare, which can lead to better management of elephants, and potentially other species, in zoological settings.

**Abstract:**

Accredited zoos and aquariums value superior animal husbandry and strive to ensure that the physical, psychological, and social needs of animals are met. In North America, the Association of Zoos and Aquariums (AZA) relies on species-specific standards to ensure facilities provide the best care for collection animals. The AZA also makes explicit recommendations for long-term monitoring of welfare. Data collected through behavioral observations can be used to modify management as animals respond over time to social, environmental, or physical changes. In long-lived, social species like elephants, it is particularly important to document herd dynamics, calf development, geriatric health, and social bonds throughout their lifetimes. The Smithsonian’s National Zoological Park housed one male and six female Asian elephants in dynamic social groupings. Behavioral observations were conducted on all elephants for two years using two methods involving ZooMonitor, closed circuit cameras, and camera traps. The goal was to compare how these two methods function to provide individual activity budgets, habitat use, and social interactions. Methodologies such as these, alone or in combination, have the potential to produce valuable data about potential changes in welfare over time in a zoological setting and can be performed either by staff or volunteers with high reliability.

## 1. Introduction

Asian elephants (*Elephas maximus*) are classified as endangered by the International Union for Conservation of Nature (IUCN) [1]. With habitat loss and increased human-elephant conflict being the main contributing factors for the decline [2,3]. One of the goals of the Association of Zoos and Aquariums (AZA) is to create healthy breeding populations to maintain species that face conservation challenges in the wild [4]. Currently, there are 138 Asian elephants at 32 AZA facilities; however, the population has never been self-sustaining because of high infant mortality and low reproductive rates [5]. Asian elephants have complex social systems that have historically been difficult to maintain in ex situ conditions [6]. Thus, AZA now requires facilities housing elephants to maintain at “least three females, two males, or three elephants of mixed sex” in order to provide species appropriate social groupings [7].

Monitoring activity levels of individual zoo elephants provides information on behavior, habitat use, and social interactions that can inform management practices to best fit the needs of elephants. In 2018, AZA added accreditation standard 1.5.0 that recognizes the need to assess welfare on an individual level, and a growing body of work has established the importance of providing quality care 24/7 [8]. Zoological facilities strive on all levels to enhance social experiences, improve opportunities for choice and control, promote species-specific behaviors, and reduce negative behaviors like stereotypies, which can be indicators of compromised welfare [6,9]. Identifying positive indicators of welfare for each species is an important step in determining where resources for improved care should be placed. For example, elephants given outdoor access overnight and that spend more time in social groups have shown decreased rates of stereotypic behaviors and lower glucocorticoids [10,11,12]. Likewise, more natural feeding and foraging behaviors are observed in elephants given smaller meals at more frequent times throughout the day, and increased feeding and foraging behaviors correspond to a decrease in aberrant behavior [13,14]. Other important indicators of positive welfare in elephants are resting and sleeping behaviors, in both standing and recumbent positions [15,16].

Performing systematic behavior observations [17] can be challenging for zoo staff taxed with the daily routines of animal care, but it is crucial to helping managers understand how factors in the zoological environment affect individual animals in real time. With increased technological advancements, there are more opportunities to observe and monitor animals, and gain a more comprehensive view of an individual’s behavior. Closed-circuit camera systems, camera traps, and GoPros are now being utilized and allow researchers and animal care staff to monitor behavior around the clock [18], and in the absence of an observer, can provide more well-rounded observations of the animals’ daily lives than ever before [19]. For example, camera traps used to monitor diurnal and nocturnal activity and space use in captive flamingos demonstrated that enclosure usage widens at night when nocturnal foraging and feeding behaviors occur [20]. Camera traps have also been used for 24 h observations of stereotypic behaviors in giraffes [21]. Giraffes exhibited increased stereotypies overnight versus during the day; however, those behaviors decreased when browse feeding enrichment was introduced. Information gained through the use of this type of surveillance allows animal managers to make simple adjustments that can increase an individual animal’s welfare. Finally, new software developments are available for data collection and tracking behavior, including Lincoln Park Zoo’s ZooMonitor application. Over 200 organizations have used ZooMonitor since its public release in 2016, providing valuable information to help managers make more informed management decisions to improve welfare [22,23].

In this study, the behavior of one male and six female Asian elephants at the Smithsonian’s National Zoological Park (NZP) was monitored by two methods. The first method utilized closed-circuit infrared cameras (CCTV) combined with camera traps to fill in missing data from CCTV blind spots in habitat areas during 24 h interval observations. The second method employed 30 min focal observations in ZooMonitor [24] six times per week on each elephant. Observations were conducted over a two-year period, and the methods compared to examine three commonly measured welfare factors in zoo-housed animals: Activity budgets, habitat use, and social interactions. We predicted that either the 30 min focal or 24 h interval observation methods would provide reliable data on these three welfare factors (activity budget, habitat use, and social interactions). The ability to accurately monitor behavior is key to determining the effectiveness of management changes and assisting animal care staff in making science-based decisions to ensure optimal welfare.

## 2. Materials and Methods

All observations took place between 6 November 2017 and 25 November 2019 in the NZP elephant exhibit, which was built in the 1930s and underwent a major renovation in 2013 to expand the indoor and outdoor space. The exhibit underwent further renovations to improve the pool feature, which was closed during the first year of the study but did not impact data collection.

### 2.1. Animals and Management

A summary of the elephant subjects is presented in Table 1. Ambika was wildborn in India, captured for work in a logging camp, and then moved to the NZP around age 12. As the eldest, she acted as a surrogate mother to Shanthi, and was almost exclusively housed with Shanthi along with Bozie, who arrived in 2013. Kamala, Maharani and Swarna came to the NZP from the Calgary Zoo in Canada as a social group and were housed together during the evenings and most days. Later, Swarna was often kept with Ambika, Shanthi, and Bozie during the day. Spike was captive-born and resided at Calgary Zoo from 1992 to 2013, then Busch Gardens Tampa Bay, before coming to the NZP. Shortly after his arrival in 2018, Spike was reintroduced during the day to Kamala, Maharani, and Swarna, with whom he had spent time at Calgary Zoo. Finally, introductions began between Bozie and Maharani starting in April 2018.

Animal care staff arrived at 06:30 h and began the day by completing general health checks, feeding the elephants, providing foot care and baths, and working on husbandry behaviors. The elephants were then rotated outside to any of four habitats at approximately 09:00 h. Habitats had interconnecting gates, providing elephants with tactile and visual contact when housed in adjacent habitats. Training demonstrations occurred at 11:00 and 14:00 h daily when keepers showed zoo visitors husbandry behaviors and discussed elephant conservation. Participation from the elephants was voluntary. Typically, overnight access was given in the mid-afternoon between 13:30–14:00 h. Before and after demos, elephants were free to explore the enclosures and engage with enrichment items (toys, browse, scents, novel objects etc.) and habitat structures such as pools, sand banks, shade structures, and feeding devices. Animal care staff managed social groupings based on compatibility and health considerations.

Diets consisted of grass hay and bamboo fed socially, and pelleted grain, produce, and vitamins fed individually during training sessions. Hay made up the largest portion of the elephants’ diet in terms of both weight and volume and was offered as multiple feedings throughout the day. Due to age and dental health, the oldest elephant (Ambika) was offered grass hay in a pre-chipped form. Otherwise elephants were offered hay from a standard bale, fed either on the ground, in elevated barrels, or secured behind 2 × 2-inch mesh.

### 2.2. Equipment

**Figure 1:**
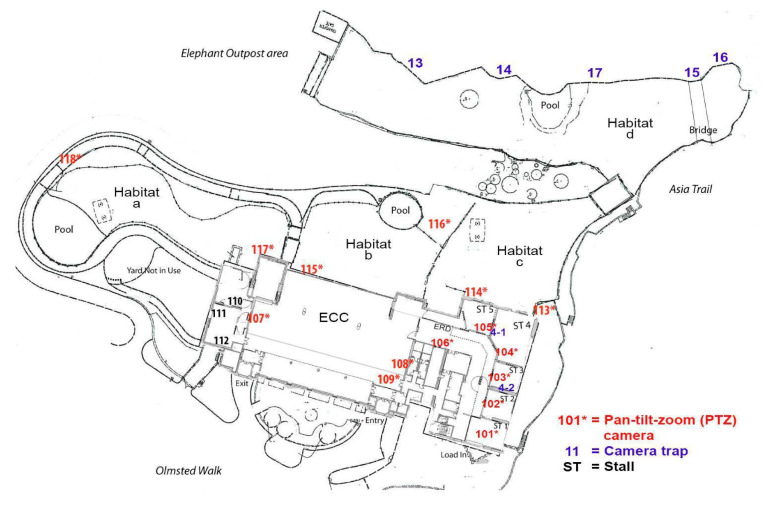
Locations of Pan-tilt-zoom (PTZ) cameras for Genetec closed-circuit system (marked in red) and camera traps (marked in blue) throughout the exhibit and stalls (ST) at the NZP. ECC refers to a large indoor Elephant Community Center with a sand floor, large pool and various feeding devices and enrichment structures and ERD is the elephant restraint device, used for training elephants for husbandry and medical procedures.

A Genetec closed-circuit infrared camera system (CCTV) (Genetec Security Center 5.9 Security Desk, Montreal, QC, Canada, www.genetec.com) was installed in February 2016 and included 18 closed-circuit cameras mounted throughout indoor and outdoor facilities (Figure 1). The cameras were pre-set at specific angles and part of a live, public-access streaming outreach venture by the NZP. Angles were occasionally modified by volunteers between the 09:00 and 16:00 h depending on where the elephants were located. The cameras were returned to the pre-set angles when the volunteers were not on site or finished for the day. Genetec recordings were available for review for 30 days after the chronicled date.

Five camera traps (Bushnell Trail Camera Trophy Cam HD 14MP Aggressor #119776C and 24 MP #119877C, Overland Park, KS, USA) were used in areas with no coverage from CCTV cameras (Figure 1). When the CCTV in Suite 4 (104) stopped working on 11 July 2018, two camera traps (4-1 and 4-2) were placed in the mezzanine above the suites to maintain coverage (Figure 1). All camera traps were set on Field Scan, Interval to record 15 s of video every 60 min on the hour. These settings did not preclude the cameras from obtaining video when the motion sensor was triggered; however, it was not used in the analyses. Additional trap settings included: Video Interval = 5 s; Night Vision Shutter = Low and Video Size 1280 × 720. All other settings remained on default. Camera traps were mounted on 2 × 4 posts cemented 0.45 m into the ground or on camera tripods on a catwalk above the indoor suites. Camera height ranged between 1.3 and 1.7 m off the ground depending on enclosure features. Once placed, the positioning did not change in order to maintain consistency in the collection. Each week, the camera trap memory cards were replaced, and batteries and settings were checked.

### 2.3. Behavioral Observations

#### 2.3.1. ZooMonitor 30 min Focal Animal Observations

**Figure 2:**
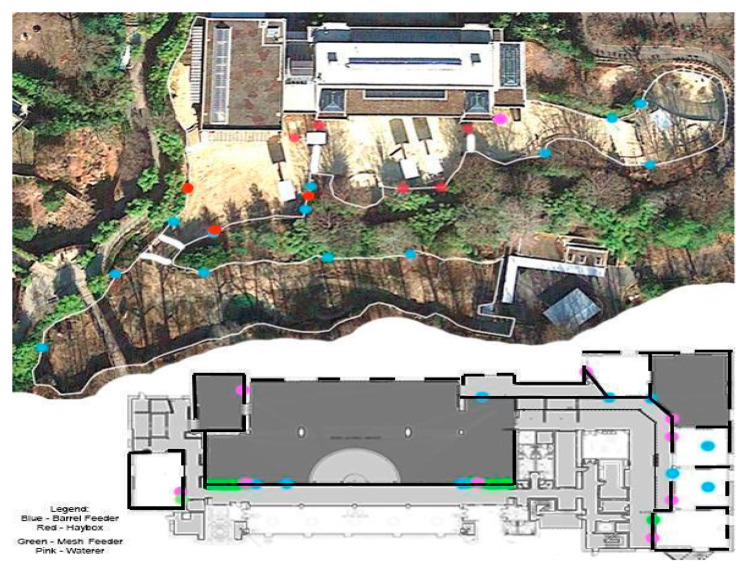
Habitat map created for ZooMonitor to specify elephant locations on 2-min intervals during behavior observations performed on 1.6 Asian elephants at the Smithsonian National Zoological Park. Colored dots represent various habitat features.

ZooMonitor Version 1 Mobile application software [23] was utilized for 30 min focal behavior observations during which state behaviors, location, and social interactions, were recorded at 2 min intervals (Table 2). A map of the indoor and outdoor habitats was created for use in ZooMonitor (Figure 2) to provide a birds-eye view and monitor habitat features, such as feeding devices and water sources. Three observations occurred at randomized times between the hours of 06:00 and 17:00 h (day) and another three between 18:00 and 5:00 (night), totaling 3 h of observations per elephant each week. Observation data were collected live with observers watching elephants directly or using the Genetec camera system to visualize multiple enclosures at one time if elephants had access to various areas. The Genetec system was also used to perform ZooMonitor observations from overnight on recorded video as it allowed observers to view video footage stored for up to 30 days. In the event of reduced animal visibility or keeper handling, times were occasionally shifted to complete the observations when elephants were visible and not being handled a majority of the observation period. Multiple elephants may have been observed at the same time, but only if they were housed together at the time of the observation. No more than four elephants were observed during any one observation period.

#### 2.3.2. Twenty-Four Hour Interval Sampling

Hourly behavior sampling was performed on two randomly selected days per week for 24 consecutive hours using both the CCTV in combination with the camera traps. These observations included data from all areas throughout the elephant indoor and outdoor habitat with camera trap footage integrated into these observations to include areas that the Genetec CCTV system did not cover. The observations of animal location, behavior and social group were performed on the hour plus 5 s (12:00:05) (See Table 2 for ethogram definitions).

#### 2.3.3. Reliability Analysis

Spearman rank correlation was used to measure inter-rater reliability among 18 with significant correlation coefficients (*p* < 0.01) for both states (r_s_ = 0.891 ± 0.024, *n* = 11) and social interaction (r_s_ = 0.916 ± 0.013, *n* = 11).

### 2.4. Data Assimilation and Analysis

#### 2.4.1. Activity Budgets

ZooMonitor 30 min focal observations (referred to as 30 min focal observations throughout this manuscript) and 24 h interval data (referred to as 24 h interval) were assimilated in Microsoft Excel (v16.42, Redmond, WA, USA) to determine the weekly proportion of time each elephant spent exhibiting a given behavior by dividing the weekly count per behavior over the total intervals observed and then averaging the data. Weekly values were chosen to more accurately represent changes over time.

Resulting activity budgets excluded “Not Visible”, and behaviors were combined into two additional categories: Sleep, and Stereotypy. Sleep incorporated “Rest” and “Recumbent.” Stereotypy included “Sway,” “Sway/Feed,” “Pace,” and “Gate Wait” (Table 2). Behavioral observations using the two methods were compared on individual state behaviors as well as these two combined behaviors.

#### 2.4.2. Social Behavior

Instances of positive, negative, and neutral interactions (Table 2) with other elephants in a cohort were collected during both 30 min focal and 24 h interval observations. Similar to analyses used to estimate elephant activity budgets, a proportion of time was calculated for each elephant by dividing the weekly counts per type of interaction over the total intervals observed and then averaging these into proportions per social interaction category.

#### 2.4.3. Habitat Use

When coding 24 h interval data, a grid number corresponding to the grid used in ZooMonitor was also recorded. The proportion of time spent in various locations around Elephant Trails was determined first for each elephant and then for all elephants over time so that seasonal trends could be examined. Next, behavioral proportion of time was calculated for each elephant based on where behaviors were taking place and resulting activity budgets were generated.

#### 2.4.4. Statistical Analyses

Data were analyzed using paired samples *t*-tests, Spearman correlation, and repeated measures analysis of variance (ANOVA) to compare observation methods (30 min focal versus 24 h interval sampling) (Table 2). A Kendall rank correlation was used on monthly habitat use data to look at seasonal patterns. A chi-squared test was used to confirm the number of observations performed per elephant. Statistical analyses were performed in SPSS v23 (IBM, Somers, NY, USA) or Statplus (AnalystSoft, v7.1.29, Walnut, CA, USA.). When examining changes in behaviors or social interactions, individual elephant’s positive, negative, and neutral interactions with another elephant were plotted timewise for linear regression analyses. Linear regressions were performed on the proportions of time that elephants spent in negative, neutral, and positive interactions, over the course of the study.

## 3. Results

### 3.1. Data Collection

Thirty-minute focal (6 per week) and 24 h interval observations utilizing CCTV along with camera trap observations (2 days per week) were performed for each of the six female elephants between November 2017 and 2019 and the bull beginning in March 2018 upon his transfer to NZP (Table 1) so fewer observations were performed on him. However, among the six elephants present at the beginning of the study, for each observation method, the number of total observations did not vary significantly (ZooMonitor: mean = 686.17 ± 5.82, range = 673–712, *n* = 4117, χ^2^ = 1.48, df = 5, *p* > 0.05; 24 h observation: *n* = 1236 total, 206 per female elephant, χ^2^ = 0, df = 5, *p* > 0.05).

### 3.2. Activity Budgets

“Feed” and “Stand” were the predominant behaviors identified in both methods, and when combined included over half of the elephants’ activity budget (Table 3). Most behaviors were positively correlated between the two sampling methods, with correlation coefficients (N = 723, r (range) = 0.089–0.732, *p* < 0.05), with the exception of two behaviors, “Other” (N = 723, r = 0.047, *p* = 0.016) and “Manipulate” (N = 723, r = 0.061, *p* = 0.099). However, when examining the mean weekly proportion of time (%) several behaviors varied (*p* < 0.05) between the methods. Other key behaviors, such as “Recumbence”, “Rest”, “Sway” and “Sway/Feed” did not vary significantly (Table 3). In addition, combined values when examining Sleep = “Rest” + “Recumbence” (N = 723, 30 min focal mean = 19.28 ± 0.50%; 24 h interval mean = 18.24 ± 0.38%, *t* (two-tailed) = 1.932, *p* = 0.54) as well as Stereotypy = “Pace” + “Sway” + “Sway/Feed” + “Gate wait” (N = 723, 30 min focal mean = 10.56 ± 0.47%; 24 h interval mean = 9.80 ± 0.0.38%, *t* (two-tailed) = 1.957, *p* = 0.51) were also measured equally regardless of method.

**Figure 3:**
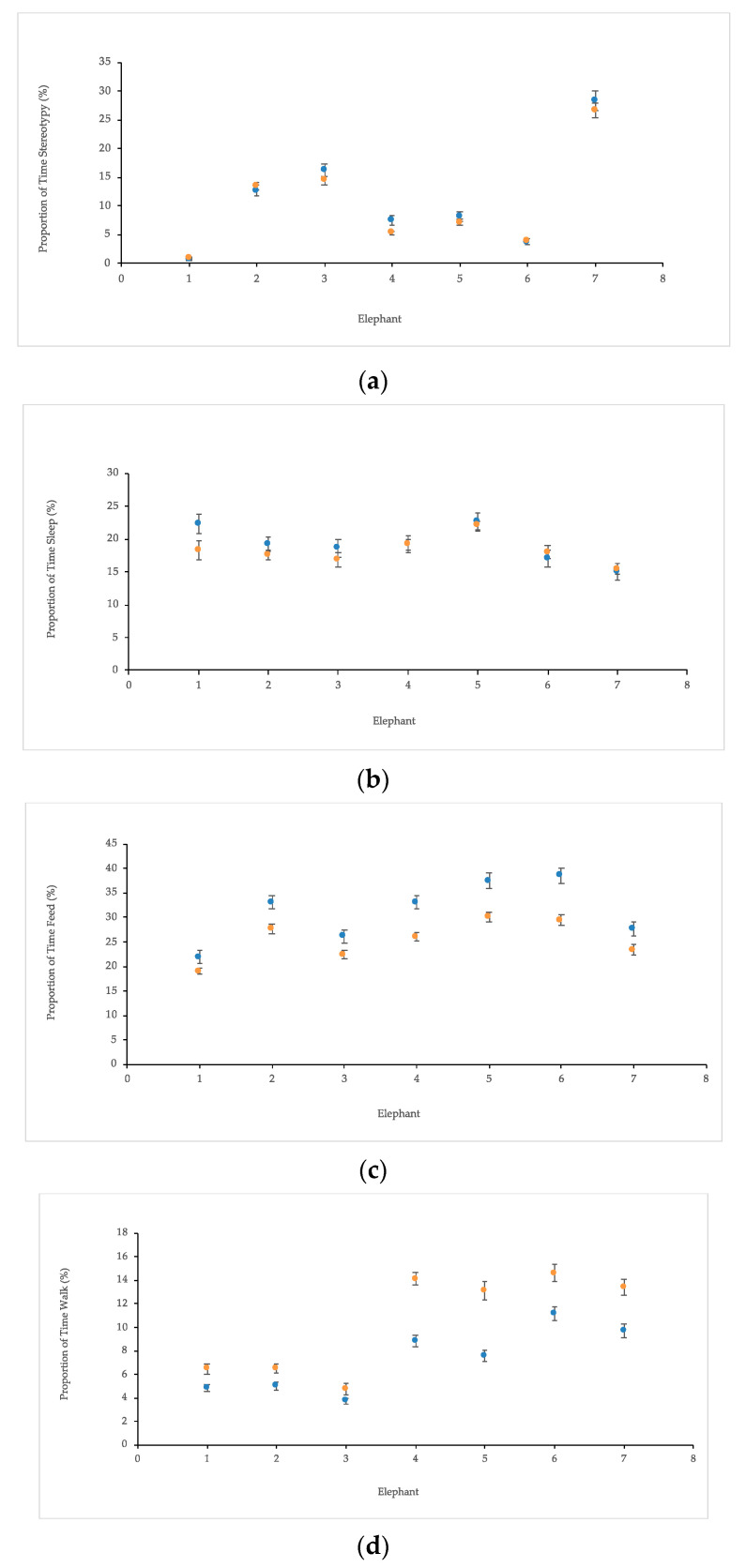
Mean (% ± standard error) proportion of time that combined behaviors (**a**) “Stereotypy” and (**b**) Sleep were observed in 1.6 Asian elephants using both 30 min focal (blue) or 24 h interval (orange) observation methods. Although (**c**) “Feed” and (**d**) “Walk” did vary, similar patterns between elephants still emerged to reveal consistent individual differences.

Individual differences between elephants were found when examining the four primary behaviors with either method (*p* > 0.05). Similar proportion of times between methods were found within elephants when looking at combined behaviors “Stereotypy” (Figure 3a) and “Sleep” (Figure 3b). However, higher rates of “Feed” were observed using 30 min focal (Figure 3c) and higher rates of “Walk” with 24 h interval (Figure 3d), though individual variations remained the same.

**Figure 4:**
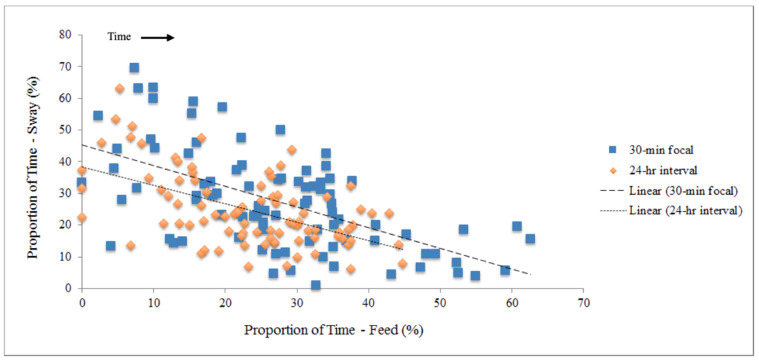
Linear regressions testing correlation of swaying and feeding behaviors observed during 30 min focal and 24 h interval sampling. Values are derived from weekly averages.

Linear regression of proportion of time observed during both observation methods revealed that there was a relationship between increased feeding and decreased swaying behavior over time in the male elephant (30 min focal: y = −0.6511X + 0.4522, R^2^ = 0.33428, *p* = 2.71 × 10^−9^; 24 h interval: y = −0.5806X + 0.3839, R^2^ = 0.28307, *p* = 6.83 × 10^−8^, Figure 4).

**Figure 5:**
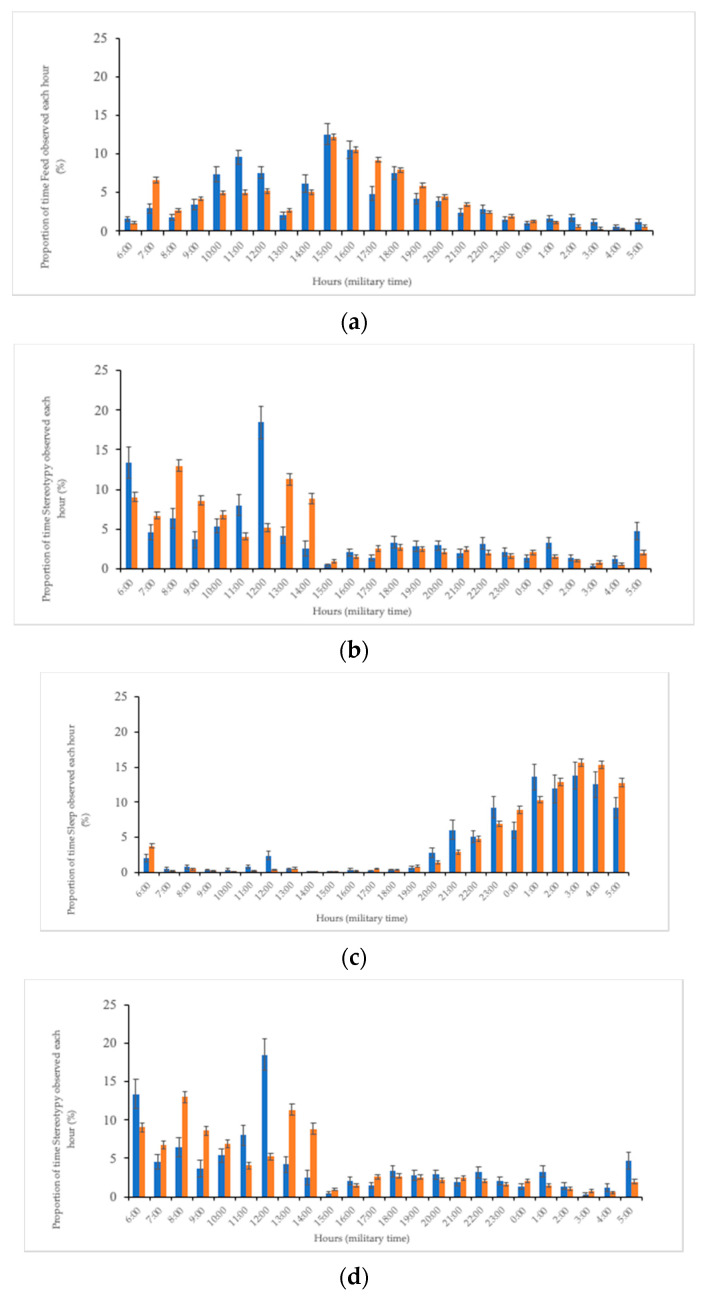
Hourly mean (% ± standard error) proportion of time for 30 min focal (blue) and 24 h interval (orange) data that elephants at the Smithsonian’s National Zoological Park (NZP) were observed performing: (**a**) Feeding behavior, (**b**) Walking behavior, (**c**) Sleeping behavior, and (**d**) Stereotypic behavior.

The weekly proportion of time for each behavior was used to calculate a percentage of time for each of four behaviors (“Feed,” “Walk,” “Sleep,” and “Stereotypy”) that were observed during that hour for either 30 min focal or 24 h interval data for each week (Figure 5). The hourly proportions of time for each week were averaged across all weeks of the study. Significant differences were found for each of the four behaviors when looking at variation in proportion of time behaviors were observed by hour; however, they tended to follow the same trends (Figure 5). Feeding behaviors were observed primarily between the hours of 07:00 and 20:00 h and then declined thereafter. There were also declines observed at 08:00 and 13:00 h when animals were generally being shifted between habitats or were being handled by animal care staff (Figure 5a). Sleeping behaviors were primarily observed at night, between 20:00 and 06:00 h (Figure 5b). By contrast, stereotypic behaviors were observed the least between 15:00 and 05:00 h, which corresponded to when the elephants received afternoon feedings and also during periods of sleep (Figure 5d). Animal care staff typically worked from 06:30–19:00 h (summer) or 06:30–17:00 h (winter). The majority of the staff left at 15:00 h each day with one person staying until 17:00 h. Some anticipatory stereotypical behavior was observed primarily during the day. Specifically, an increase in stereotypic behavior at 12:00 h corresponded with animal care lunch breaks when elephants were anticipating their return for afternoon habitat maintenance and training sessions.

### 3.3. Social Interactions

Weekly proportion of time each elephant spent with other individuals in neutral, positive, or negative social interactions was calculated from 30 min focal observations (Table 4). Negative social interactions overall were low (range = 0.03–1.59%) and were primarily observed between Bozie and Swarna. Female elephants routinely housed together spent a majority of their time greater than one body length away from each other, represented by the “None” category. Spike, the bull elephant, spent the least amount of time in social interactions, possibly because he was still adjusting to the new facility and being introduced to the females.

Maharani’s interactions with the rest of the herd were examined on an individual basis to more closely monitor her introductions with Bozie (Table 5). *t*-tests were performed on the ZooMonitor data to compare the social relationships between Maharani and other members of her primary social group, Kamala and Swarna, as well as Bozie, to whom she was introduced, over the observation period. Ambika and Shanthi were excluded from this matrix because of Maharani’s low interaction frequency (m = 0.32 ± 0.16%, N = 105). Maharani spent a greater proportion of time in negative interaction with Bozie than with Swarna (t(two-tailed) = 1.97, df = 208, *p* = 0.027), but not with her mother Kamala (*t* (two-tailed) = 1.97, df = 208, *p* = 0.53). Conversely, Maharani spent less time in positive interactions with Bozie than either Swarna: *t* (two-tailed) = 1.97, df = 208, *p* = 0.00054, or Kamala: *t* (two-tailed) = 1.97, df = 208, *p* = 0.00002). In comparison, Kamala and Swarna spent a similar proportion of time interacting negatively and positively with Maharani (Negative: *t* (two-tailed) = 1.97, df = 208, *p* = 0.97, Positive: *t* (two-tailed) = 1.97, df = 208, *p* = 0.29).

**Figure 6:**
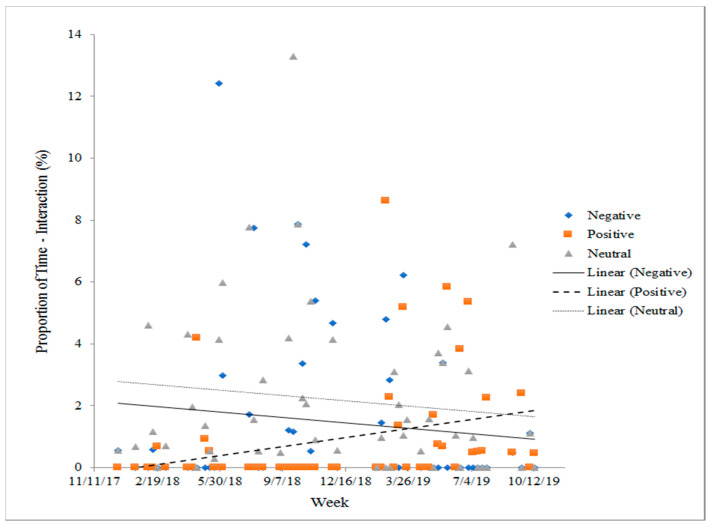
Each point represents the weekly combined proportion of total time that Bozie and Maharani interacted positively, negatively, or neutrally with each other, documented using 30 min focal observations. A significant increase in positive social interactions is noted over time as introductions continue between these two females.

Data collected in ZooMonitor showed that negative and neutral interactions between Maharani and Bozie decreased slightly (negative: y = −0.00002X + 0.66742, R^2^ = 0.01202, *p* = 0.43; neutral: y = 0.00001X + 0.6240, R^2^ = 0.01057, *p* = 0.46, Figure 6), however positive interactions between the two increased significantly (positive: y = 0.00003X − 1.229, R^2^ = 0.09293, *p* = 0.025) confirming their developing social bonds (Figure 6). Similar trends were not measured using the 24 h interval data. Although no significant changes were detected, a slight increase in neutral and positive interactions and a decrease in negative were measured through this method (neutral = y = 6 × 10^5^X − 2.506, R^2^ = 0.013, *p* = 0.326; positive: y = 0.0001X − 5.3972, R^2^ = 0.010, *p* = 0.393; negative: y = −0.0003X + 15.24, R^2^ = 0.037, *p* = 0.098).

### 3.4. Habitat Use

**Figure 7:**
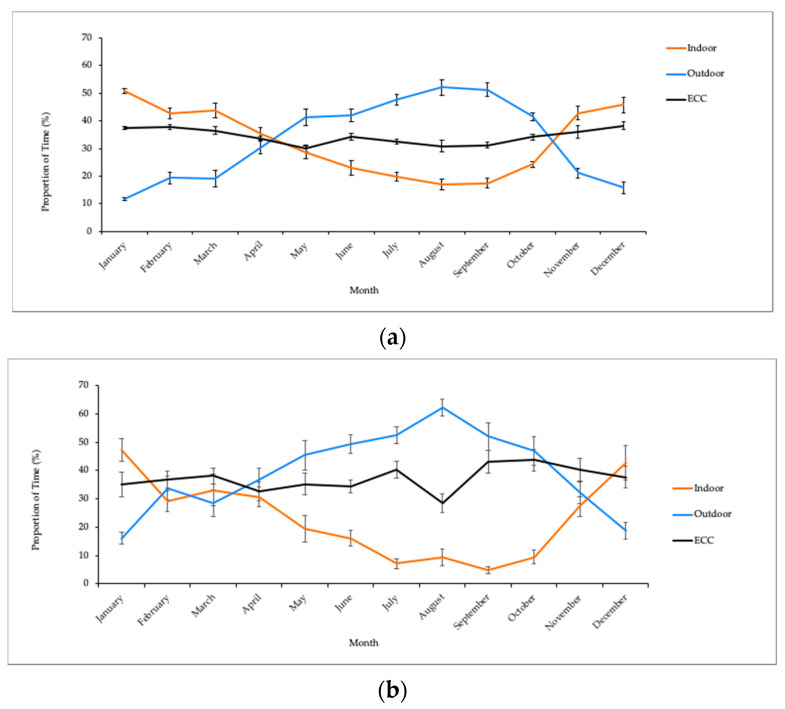
Mean (% ± standard error) monthly proportion of time in habitat use by the Smithsonian National Zoo’s elephant herd, recorded using (**a**) the 24 h closed circuit observation method or (**b**) ZooMonitor 30 min focal observations. Indoor monthly total proportion of time represents time that the elephants spent in suites 1–7. Outdoor is the monthly total proportion of time spent in habitats a–d. “ECC” refers to the Elephant Community Center, a large indoor space that opens up to habitat b.

**Figure 8:**
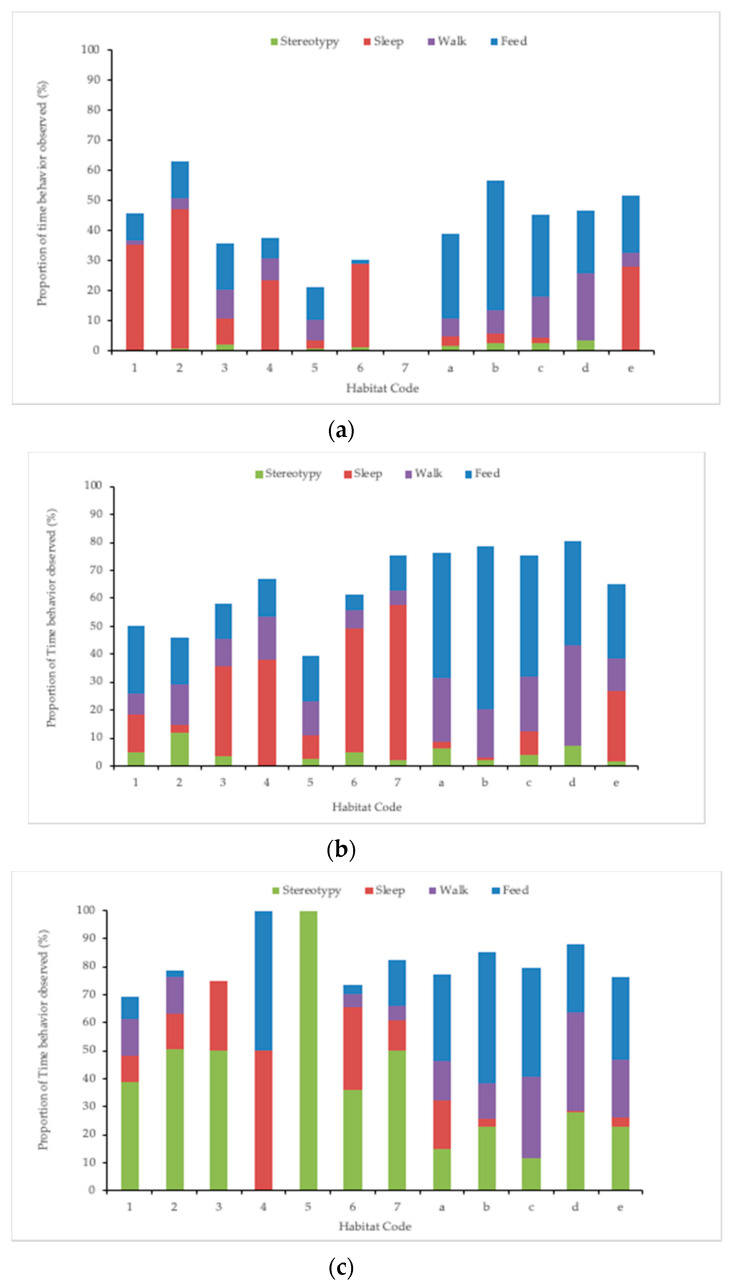
Activity budget representing proportion of time spent exhibiting four key behaviors: “Stereotypy”, “Sleep”, “Walk”, and “Feed”. Observations made on three elephants through 24 h interval monitoring are represented and include: (**a**) Ambika, (**b**) Swarna, and (**c**) Spike. Habitats include indoor suites 1–7 and Elephant Community Center and outdoor habitats a–e.

Examining data from the 24 h interval method, the herd was found to spend more time indoors than outdoors during the winter months, January–March (*p* < 0.05, w = 0.563–0.901, Figure 7a). Conversely, throughout the rest of the year, elephants spent more time outdoors or in the ECC with outdoor access (*p* < 0.05, w = 0.766–1.00, Figure 7), with the exception of April–June when they exhibited indiscriminate presence across all habitats (w = 0.033–0.246, Figure 7a). The ZooMonitor observation method generated similar outcomes, with elephants spending more time indoors in January (*p* < 0.05, w = 0.704, Figure 7b), and more time outdoors from June-October, as well as in December (*p* < 0.05, w = 0.531–1.00, Figure 8). No differences in habitat use were found in 30 min focal data for the months of February–May and November (w = 0.04–0.328, Figure 7b). ECC use remained relatively constant (mean range = 30.05–37.69%) throughout the year for both methods. Other locations, including the Elephant Restraint Device, Paddock, and Annex were excluded from this analysis because they only accounted for 0.00–0.59% of the overall habitat use budget.

Habitat use data were combined with activity budgets to identify the locations of key behaviors such as “Stereotypy”, “Sleep”, “Feed”, and “Walk” for each elephant. Spike was observed to spend more time exhibiting stereotypies in Suites 1–7 than in the habitats or ECC. Spike and Bozie exhibited stereotypic behavior more often indoors than outdoors, whereas Shanthi exhibited stereotypic behavior nearly indiscriminately across habitats (Figure 8a–c).

**Figure 9:**
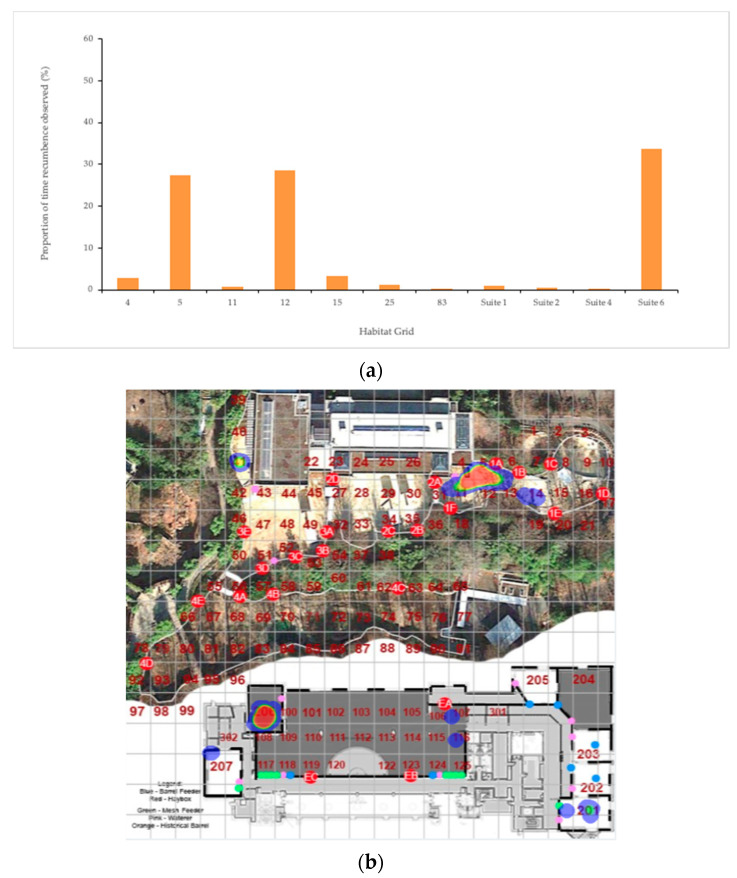
Heat map of behavior observations from (**a**) 24 h interval data and (**b**) ZooMonitor 30 min focal observations indicate that the bull elephant exhibits a preference in areas used to exhibit “Recumbence” or lay down. In (**b**), the red areas indicate more frequently observed behaviors, in this case, recumbence. Circles of various colors denote exhibit food/water features.

**Figure 10:**
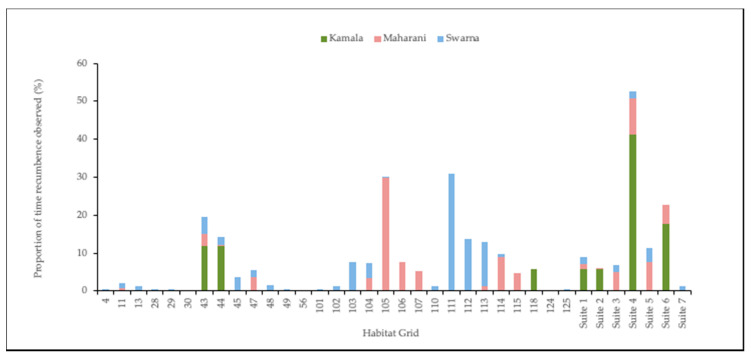
Distribution of the behavior “Recumbence” throughout the habitat of a single social group. Clear separations were observed in preferred sleeping areas and noticeable trends in sleep proximity within adjacent areas were observed among elephants.

Recumbence data were examined to determine most commonly used areas for each individual elephant to lay down (Figure 9). Spike exhibited clear sleeping location preferences that emerged in both the 24 h interval (Figure 9a) and the ZooMonitor habitat heat map export (Figure 9b). Greater proportions of time were measured in suite 6 and grids 5 and 12, located in Habitat a. Figure 10 details how a single social group, Kamala, Maharani, and Swarna distributed themselves to sleep in adjacent, but rarely overlapping areas, with distinct areas emerging that were dominated by particular individuals.

## 4. Discussion

Obtaining behavioral information on animals held within zoological facilities on a routine basis for longitudinal evaluation can be invaluable when determining successful management strategies to increase welfare. However, assessing welfare using behavioral indicators can be a time-consuming and difficult task when combined with the other husbandry duties of animal care. Furthermore, adapting behavioral observation methods to the resources available at each institution can be a challenge. The methods evaluated in this study, 30 min focal observations and 24 h interval data collection, presented two viable options for long-term, in-depth behavioral assessments of elephant activity budgets, habitat use, and social dynamics in a herd of 1.6 elephants at the NZP. Findings suggest either method can be used to determine accurate activity budgets or habitat use, but 30 min focal observations through ZooMonitor served to better describe changes in social interactions over time. Habitat use can be measured using either method

ZooMonitor is a continually updated, free, behavior observation app with multiple uses [23]. A newly added interobserver reliability feature increases the ease of maintaining high-quality systematic behavior observations for long-term assessment. Furthermore, the application interface is intuitive and streamlined, making it easy to record both positive (species-specific) and negative (stereotypic) behavioral indicators. The interval method employed through the ZooMonitor platform allowed for continuous evaluation minute to minute, which resulted in a more nuanced understanding of the behavior of elephants at NZP as described for other zoo species [23,25]. Another advantage of ZooMonitor was increased flexibility using live, in-person observations, or CCTV footage when live observations were not feasible.

Although CCTV can be expensive, having the system throughout most of Elephant Trails facilitated a more holistic evaluation of behavior through both 30 min focal and 24 h interval methods, and allowed observations of elephant interactions and activities throughout the day and night without observer effects. The combination approach of using CCTV and camera traps to accomplish the 24 h interval data collection also proved successful. Camera traps filled in areas where CCTV did not cover and were utilized on their own in some of the smaller habitats that required surveillance.

While camera traps have been used for decades to document the wild ecology of species in their native habitat [26], few studies have utilized camera traps for systematic behavior monitoring of collection animals in zoos [20,21]. For elephants, which are known to only sleep sporadically for 3-6.5 h each night [27,28] and have increased social interactions throughout the evening hours [29], 24/7 monitoring is essential to assessing welfare and informing management decisions. Camera traps have several advantages in that they are relatively inexpensive, can be secured in many different areas, and are easily moveable to answer research or husbandry questions. For example, CCTV did not cover some areas where the bull was believed to spend time laying down, so camera traps were positioned to provide this information. Camera traps also allowed for consistent 24/7 observations while observers were absent, reducing observer effects. Social interactions between recently introduced pairs like Bozie and Maharani were obtained at higher resolution with overnight monitoring, which proved crucial in obtaining a complete picture of the elephants’ behavioral repertoire and assessing social group dynamics. The applications of 24/7 surveillance are clearly beneficial and would be even more so for species that exhibit even higher nocturnal activity.

There are a few drawbacks to using CCTV and camera trap technology. Camera traps can sometimes result in low levels of visibility or lower accuracy with individual identification [19]. The camera traps used at NZP required frequent battery changes (monthly) and could be somewhat unreliable with settings changing automatically or batteries died more frequently than expected, especially in cold weather conditions. To address these shortcomings, data collection was limited to only two days a week, which allowed observers to eliminate days when the camera traps malfunctioned and did not impact the overall results. CCTV cameras were controlled by volunteers during the day, which sometimes led to poorly positioned cameras in blind spots where elephants were “Not Visible” during interval sampling. As a result, camera position presets were created to help ensure that volunteers did not leave cameras at poor visibility angles overnight. For institutions that do not have the capacity to set up CCTV, camera traps can provide invaluable information on the behavior of individual animals.

### 4.1. Activity Budgets

Activity budgets created from both methods identified individual differences among the elephants, which often confirmed animal care staff perceptions. Across both 30 min focal and 24 h interval methods, the eldest female, Ambika, was observed to spend the least amount of time feeding, whereas Spike spent the least amount of time sleeping, and Swarna spent the most amount of time walking around the enclosure. The agreement between observation styles in pinpointing these individual differences suggests that either method can be used to create activity budgets and inform on welfare states. Generally, the activity budgets obtained from the seven elephants corresponded with previous studies of the species. Feeding behaviors averaged 31.19 ± 0.58 (30 min focals) and 25.51 ± 0.37 (intervals) percent of the time observed, similar to other zoo housed elephants (27.4–41.4% daytime range in [14]).

Although both methods produced similar activity budgets, focal observations and 24 h interval observations produced slightly different rates of occurrence for certain behaviors. For example, the behaviors “Dust”, “Feed”, “Gate Wait”, “Other”, “Rest”, and “Not Visible” were observed in higher proportions through focal observations. Although 30 min focal observations were split evenly and times were randomized, observations were performed only when the elephants were expected to be visible for the majority of the 30 min observation period. Conversely, the 24 h interval sampling was conducted at the top of every hour, regardless of visibility. Higher rates of “Not Visible” were recorded via the 24 h interval method and the proportions of time spent exhibiting short-duration or event behaviors were consequently lower. Studies focused on self-care, grooming or enrichment use would be better served using a focal observation method, and should incorporate some all occurrence measures of short-duration behaviors as well [30].

When considering the impact of management on the welfare of individuals within zoological collections, both focal and interval methods reliably documented significant shifts in behaviors. For example, increases in species-specific behavior (i.e., “Feed”) and decreases in aberrant behavior (i.e., “Sway”) were observed in Spike following his transfer to NZP, as he began to utilize more habitat throughout the trail. Similar decreases in stereotypic behavior have been noted with increased feeding opportunities in other zoo housed elephants [14]. Generally, elephants housed in the collection at NZP had significantly lower rates of stereotypical behavior than those reported overall for the AZA Asian elephant zoo population (15.5–24.8% in [10]). Stereotypies overnight were not commonly observed, most likely due to the common management practice of providing elephants with indoor and outdoor access for most of the year; a practice that has also been shown to decrease stereotypies [13] and be associated with lower fecal glucocorticoid metabolite concentrations [11]. The high correlation in behavior data between focal and interval collection methods in this study suggests both can be used to evaluate responses of individual elephants to zoo transfers and exhibit design changes to meet targeted behavior goals.

The two methods might also be asymmetrically suited for different study species. For example, 24 h interval observations work best for socially housed species or in animals held in larger numbers, where 30 min focal observations can be time prohibitive if there are many individuals to observe. Furthermore, 24 h data provide a more complete picture of an animal’s behavior throughout the day. If focal observations are not possible due to handling or poor visibility, or overnight when there are no observers, camera traps can be deployed to supplement the 24 h interval data.

### 4.2. Social Interactions

Social behavior data collected through 30 min focal sampling in ZooMonitor proved to be more useful towards the goal of obtaining high-resolution and comprehensive interaction data during introductions between new individuals. Social dynamics within the female cohort of elephants at the NZP, as well as interactions within smaller groupings of elephants like Bozie and Maharani, or the bull elephant Spike and the females, could be documented at high resolution, during times when the animals were actively housed together.

Unlike the 24 h interval sampling, in which observations happened at the top of each hour, 30 min focal sampling in ZooMonitor provided more condensed time to observe the social context between elephants when rating relationships at each point. The frequency of collection during periods of potentially short introductions done throughout the day allowed for additional statistical power to inform social changes such as those observed with Maharani and Bozie’s increased positive interactions as introductions continued. For animals housed together around the clock, the 24 h provides reliable information on relationships, however for monitoring introductions or specific social changes increasing the frequency of the 24 h interval data collection would most likely result in more accurate social information.

### 4.3. Habitat Use

Habitat use was best described using 24 h interval observations, as camera trap data filled in area gaps that the closed-circuit cameras did not cover or when CCTV cameras occasionally went offline for scheduled updates or momentary disconnection. Therefore, resulting data were collected across a much more consistent view of the entire habitat area, suggesting that camera traps are a reliable technology for comprehensive, 24/7 surveillance of animals in zoological facilities that cannot install CCTV [20].

Both methods showed that habitat use followed a typical seasonal trend. The NZP’s indoor ECC and suites were equipped with a robust heating system using both steam-fed forced air and a geothermal system that heated the floors and walls. As a result, elephants were given access to outdoor habitat throughout the night even when the temperature conditions fell below the indoor minimum (12 °C). Some older elephants showed a preference for warmer conditions and minimum lock-in temperatures were adjusted as needed for them. Occasionally, during summer evenings, elephants were sometimes not given overnight, indoor access, a management strategy found to increase positive species-specific behaviors [30].

Specifically, when examining recumbence, it was observed that one group of females, Kamala, Maharani, and Swarna exhibited recumbence outdoors primarily in Habitat c, but only when they were locked outside with no access to indoor suites or the ECC. Outside of that time, Kamala and Swarna would only lay down when housed individually overnight. Both methods reliably pinpointed where and how frequently certain behaviors occurred within the habitat. In addition, elephants with joint disease, Ambika and Shanthi, did not exhibit recumbence, which aligns with previous studies [16]. All the other elephants exhibited recumbence on average 7% of their time (~3.5 h per night), primarily between the hours of 21:00–05:00 h, with highest rates of recumbence falling between 24:00–04:00 h. This pattern closely follows previous reports in zoo Asian elephants [15]. Recumbence in elephants is a key behavior that is essential to their overall welfare. In addition to decreasing with the onset of joint disease, recumbence also declines when environmental or social stressors are present [16]. In those cases, management changes must be implemented to allow for adequate periods of rest to avoid “episodic collapse” or “excessive drowsiness” [15].

In this study, it was observed that areas most frequently chosen for laying down consisted of sand floors, demonstrating a preference for that substrate over grassy areas and rubberized floors, similar to what has been previously reported in this species [16].

Zoological institutions can efficiently address animal needs and improve welfare by utilizing either of these demonstrated, feasible options to perform long-term behavioral assessment and monitor changes within their collection animals. Both observation methods were successful at documenting elephant activity changes overtime and could be used to inform welfare management decisions. The 24 h interval observations measured more consistently across the day and night by incorporating the field scan feature of camera traps so that every hour was represented equally. This observation style might be more appropriate for investigating behavioral activity that may be influenced by visitors or staff, or for larger habitats that may be expensive or too remote to outfit with CCTV. By contrast, 30 min focal sampling in ZooMonitor allowed for a higher-resolution and contextual understanding of social interactions and this software program allows for researchers to monitor interval, event, or continuous behaviors, which can help answer more intricate research or management questions. Broad-spectrum research initiatives may benefit from utilizing both sampling methods and combining them to create more comprehensive studies. However, for the purpose of maintaining appropriate levels of welfare monitoring on individual collection animals, either of these methods can provide a simplistic way to incorporate round the clock behavioral information for ex situ management.

## 5. Conclusions

Zoos and aquariums can use simplistic behavioral observation methods to obtain information on activity budgets, habitat use, and social interactions that can inform management decisions and improve welfare of animals in their care. Utilizing multiple observation technologies, such as closed-circuit infrared cameras, camera traps, and programs like ZooMonitor can provide consistent behavioral information 24/7 on collection animals while minimizing time and resources typically necessary for long-term monitoring.

## Figures and Tables

**Table 1:** Life history summary of the seven Asian elephants evaluated in the study at the Smithsonian National Zoological Park (NZP).

Name	Sex ♂/♀	Studbook #	Birth Year	Origin	Arrival at NZP	Weeks Observed
Bozie	♀	77	1975	Sri Lanka	2013	106
Ambika	♀	166	1948	India	1961	106
Shanthi	♀	165	1975	Sri Lanka	1976	106
Maharani	♀	307	1990	Calgary Zoo	2014	106
Kamala	♀	145	1975	Sri Lanka	2014	106
Swarna	♀	146	1975	Sri Lanka	2014	106
Spike	♂	141	1981	Zoo Miami	2018	89

**Table 2:** Ethogram of behaviors observed between November 2017 and 2019 on one male and six female Asian elephants housed at the Smithsonian National Zoological Park. During data collection, the location of each elephant was also recorded.

Category	Behavior	Definition
Social Interaction	None	Elephants are not within one body length of other elephants
	Neutral	Elephants are within one body length, but engaged in separate behaviors and not interacting in any way (i.e., one sleeping and the other standing within one body length)
	Positive	Elephants are within one body length of each other and are engaging in positive social behaviors (i.e., food sharing/feeding from same feeder, trunk touching/investigating, following, playing, etc.)
	Negative	Elephants are within one body length of each other and are engaged in negative social behaviors, including any aggression (i.e., head butting, pushing, backing into each other, biting, displace, block, chase, herd, etc.)
State Behaviors	Walk	Elephant is traveling on their own through the exhibit at least 2 body lengths, excludes movement due to social context or movement with food; includes walking or running
	Stand	Elephant is stationary, but alert and actively using trunk to sniff or investigate environment (ground, trees, etc.); excludes manipulate and gate wait
	Recumbent	Elephant laying on their side to rest, either flat on the ground or up against a sand bank for support
	Feed	Actively feeding on anything either provided or naturally occurring in exhibit—includes picking up, manipulating and/or consuming hay or browse distributed by keepers or dropped by other elephants, also includes carrying hay from one location to another
	Manipulate	Elephant uses trunk, legs, or body to play or investigate enrichment or other non-permanent items that are also non-food/browse related; trumps stand
	Dust	Elephant throw sand, dirt, water on back
	Water	Elephant uses trunk to toss water onto its body or submerges all or part of their body into the water; includes playing with enrichment items in or with water (includes drinking); trumps manipulate
	Sway/Feed	In combination, an elephant shifts its weight along a horizontal-perpendicular axis to the elephant’s spine from leg to leg WHILE actively feeding—Add feeding modifier; Record if elephant has restricted access to either habitats or building; trumps gate wait if animal is sway/feed at a door
	Sway	Elephant shifts its weight along a horizontal-perpendicular axis to the elephant’s spine from leg to leg; Record if elephant has restricted access to either yards or building; trumps gate wait if animal is swaying at a door
	Gate Wait	Elephant is standing (within 1 body length) of a door or yard gate that is closed and is facing and focused as if to walk through—includes leaning their head in or sniffing/touching door with trunk; trumps stand
	Pace	Elephant travels the same path at least three times, can include travel back and forth or in a specific route tracing pattern
	Rest	Leaning, resting, sleeping, stationary; excludes recumbent or any other behavior where active manipulation, exploration, feeding, or social activity is occurring
	Other	Elephant is performing another behavior not listed above (i.e., self-maintenance, masturbation, etc.)
	Not Visible	Out of sight or bad observation

**Table 3:** Mean (% ± standard error) proportion of time elephants were observed exhibiting state behaviors through 30 min focal observations (ZooMonitor) versus 24 h interval closed circuit camera with camera trap sampling methods. Results were compared between methods using a paired sample *t*-test.

Behavior	ZooMonitor		24 h Interval		*t*-Test		
	N	%	N	%	t	df	*p*
Dust	723	1.32 ± 0.11	723	1.23 ± 0.11	0.795	722	0.43
Feed *	723	31.19 ± 0.58	723	25.51 ± 0.37	9.818	722	0.00
Gate Wait *	723	1.07 ± 0.09	723	1.59 ± 0.10	−3.982	722	0.00
Manipulate	723	0.66 ± 0.06	723	0.56 ± 0.05	1.341	722	0.18
Other *	723	0.59 ± 0.04	723	0.44 ± 0.05	2.49	722	0.01
Pace *	723	2.09 ± 0.21	723	1.24 ± 0.11	4.062	722	0.00
Recumbent	723	7.51 ± 0.44	723	7.21 ± 0.33	0.86	722	0.39
Rest	723	11.77 ± 0.51	723	11.02 ± 0.45	1.665	722	0.10
Stand *	723	27.62 ± 0.58	723	33.08 ± 0.57	−8.991	722	0.00
Sway	723	7.33 ± 0.46	723	6.92 ± 0.38	1.296	722	0.20
Walk *	723	7.20 ± 0.19	723	10.32 ± 0.27	−11.403	722	0.00
Water *	723	1.58 ± 0.11	723	0.82 ± 0.06	6.522	722	0.00
Sway/Feed	723	0.08 ± 0.02	723	0.06 ± 0.02	0.957	722	0.34
Not Visible *	723	5.31 ± 0.25	723	16.09 ± 0.42	−23.289	722	0.00

Significance is denoted next to behavior using an asterisk (*). N refers to the number of weeks, df is the degrees of freedom. Observations of “Not Visible” were removed from calculations of each behavior, however the mean proportion of time “Not Visible” was observed is reported below to demonstrate differences in animal visibility during different observation methods.

**Table 4:** ZooMonitor (30 min focal) data (% ± standard error) for social interactions per elephant, rated on each two-minute interval as “Negative”, “Positive”, “Neutral” if elephants were within one body length of each other or “None”. Mean weekly proportion of time for each type of social interaction was determined for N number of weeks.

ZooMonitor	Negative		Positive	Neutral	None
	N	%	%	%	%
Bozie	105	1.59 ± 0.30	4.46 ± 0.54	35.95 ± 1.55	58.00 ± 1.52
Ambika	105	0.10 ± 0.04	4.34 ± 0.53	34.03 ± 1.56	61.53 ± 1.55
Shanthi	105	0.03 ± 0.02	6.24 ± 0.71	44.76 ± 1.58	48.96 ± 1.48
Kamala	105	0.81 ± 0.17	3.50 ± 0.51	21.30 ± 1.26	74.38 ± 1.30
Maharani	105	1.17 ± 0.22	5.46 ± 0.75	30.26 ± 1.35	63.11 ± 1.31
Swarna	105	1.52 ± 0.22	3.06 ± 0.50	24.41 ± 1.29	71.01 ± 1.22
Spike	89	0.02 ± 0.02	1.54 ± 0.40	3.92 ± 0.64	94.52 ± 0.83

**Table 5:** Mean (% ± standard error) proportion of time that Maharani was involved in social interactions with other elephants during ZooMonitor 30 min focal observations. Mean weekly proportion of time for each type of social interaction during N number of weeks.

ZooMonitor	Negative		Positive		Neutral	
	N	%	N	%	N	%
Bozie	105	0.74 ± 0.20	105	0.47 ± 0.13	105	1.11 ± 0.21
Kamala	105	0.28 ± 0.12	105	2.44 ± 0.43	105	14.90 ± 1.10
Swarna	105	0.28 ± 0.06	105	1.84 ± 0.37	105	16.98 ± 1.19
Spike	89	0.01 ± 0.01	89	2.23 ± 0.47	89	1.09 ± 0.39
